# Do Social Conditions Affect Capuchin Monkeys’ (*Cebus apella*) Choices in a Quantity Judgment Task?

**DOI:** 10.3389/fpsyg.2012.00492

**Published:** 2012-11-16

**Authors:** Michael J. Beran, Bonnie M. Perdue, Audrey E. Parrish, Theodore A. Evans

**Affiliations:** ^1^Language Research Center, Georgia State UniversityAtlanta, GA, USA; ^2^Psychology Department, Georgia State UniversityAtlanta, GA, USA

**Keywords:** quantity judgments, uncertainty, social testing, competition, capuchin monkeys, *Cebus apella*

## Abstract

Beran et al. (2012) reported that capuchin monkeys closely matched the performance of humans in a quantity judgment test in which information was incomplete but a judgment still had to be made. In each test session, subjects first made quantity judgments between two known options. Then, they made choices where only one option was visible. Both humans and capuchin monkeys were guided by past outcomes, as they shifted from selecting a known option to selecting an unknown option at the point at which the known option went from being more than the average rate of return to less than the average rate of return from earlier choices in the test session. Here, we expanded this assessment of what guides quantity judgment choice behavior in the face of incomplete information to include manipulations to the *unselected* quantity. We manipulated the unchosen set in two ways: first, we showed the monkeys what they did not get (the unchosen set), anticipating that “losses” would weigh heavily on subsequent trials in which the same known quantity was presented. Second, we sometimes gave the unchosen set to another monkey, anticipating that this social manipulation might influence the risk-taking responses of the focal monkey when faced with incomplete information. However, neither manipulation caused difficulty for the monkeys who instead continued to use the rational strategy of choosing known sets when they were as large as or larger than the average rate of return in the session, and choosing the unknown (riskier) set when the known set was not sufficiently large. As in past experiments, this was true across a variety of daily ranges of quantities, indicating that monkeys were not using some absolute quantity as a threshold for selecting (or not) the known set, but instead continued to use the daily average rate of return to determine when to choose the known versus the unknown quantity.

## Introduction

Individuals from many species are commonly faced with making decisions between two or more mutually exclusive options, particularly when it comes to foraging decisions and the attempt to maximize the amount of food one can get while minimizing the effort required and minimizing the risk that no food will be obtained. In one of the least risky, but more prevalent laboratory situations that is presented, organisms must choose between two quantities, and those individuals who are best at discriminating carefully between the choices and picking the larger one will net the greatest benefit. Perhaps unsurprisingly, many species are quite adept at making such relative quantity judgments (for an overview, see Brannon and Roitman, 2003). These species include insects (Chittka and Geiger, 1995; Dacke and Srinivasan, 2008), fish (Agrillo et al., 2007, 2008; Pfifer et al., 2012), amphibians (Uller et al., 2003; Krusche et al., 2010), birds (Emmerton, 1998; Rugani et al., 2008), and many mammals including voles (Ferkin et al., 2005), dogs (Ward and Smuts, 2007), bears (Vonk and Beran, 2012), elephants (Irie-Sugimoto et al., 2009; Perdue et al., 2012), marine mammals (Kilian et al., 2003; Jaakkola et al., 2005; Abramson et al., 2011), and non-human primates (e.g., Call, 2000; Beran, 2001, 2004, 2012; Anderson et al., 2005, 2007; Hanus and Call, 2007; Tomonaga, 2007; Addessi et al., 2008; Evans et al., 2009).

Recent work in our lab has shown a strong consistency across species in dealing very adaptively with uncertain or incomplete information in a quantity judgment task. Beran et al. (2009) devised a test in which chimpanzees first performed 15 trials in which they always saw each of two sets of food items, and then chose between them when they were covered. As expected, the chimpanzees were consistent in choosing the larger set. The critical test occurred during the second block of 15 trials in each session, when only one set was revealed, whereas the other remained unknown at the point of choosing. The chimpanzees responded in that case by basing their choice (or avoidance) of the unknown quantity on the amount of food in the known quantity. When the known amount was close to, or exceeded, the average quantity of items obtained across the first 15 trials, the chimpanzees selected the known set. But, if the known amount was smaller than the average, they took the risk of choosing the unknown set. This strategy occurred across a range of quantities tested across different days, and so the chimpanzees showed great flexibility in their application of this heuristic for dealing with incomplete information. In a second study Beran et al. (2012) directly compared another primate species, the capuchin monkey, with adult humans, and the same result occurred in both of these groups, providing a strong convergence of evidence that multiple primate species seem to keep a running tally of how well they have been getting rewarded at the task, and can use that information when it might be informative.

Despite this clear evidence of flexible responding in the face of incomplete information, there remain a number of questions about whether participants would sustain this kind of responding under different conditions. One can imagine that certain circumstances may produce a stronger drive to select the known set over the unknown set even when the known set is smaller than the average, for example if the unknown set involved a large degree of risk. One could manipulate risk by using conditions that kept shifting the average rates of return during the training trials, and one could manipulate the potential for extreme gains and losses for either taking or not taking the known set when faced with an unknown option. These manipulations would allow one to determine how robust the heuristic of using the ongoing representation of averages in quantity assessments is, or whether it is sensitive to fluctuations and extremes in quantity judgment.

Another likely candidate for disrupting the patterns of responding found previously would be the introduction of a more competitive circumstance. Often, putting animals in more competitive versus less competitive situations can change the nature of their responding to various tasks. For instance, rhesus monkeys have shown a speed-accuracy trade-off when directly competing against a partner in a computerized paradigm in comparison to working alone, suggesting a shift in individual strategy in response to the altered social nature of the task (Washburn et al., 1990). Moreover, both chimpanzees and rhesus monkeys have shown sensitivity in reasoning about another’s potential visual knowledge when placed in a situation where competition over food sources was likely (e.g., Hare et al., 2001; Flombaum and Santos, 2005) but failed to show this same sensitivity to a conspecific’s perceptual cues in non-competitive tasks (e.g., Tomasello and Call, 1997; Povinelli, 2000). Primates also appear to be highly sensitive to the mere likelihood for competition and alter their expression of knowledge states in the presence of higher-ranking individuals (Drea and Wallen, 1999). Thus, we tested whether such effects might emerge within our quantity judgment task.

In a standard competitive task, a salient component would be the loss of food to a conspecific, either due to direct competition over the food source or monopolization of the source by a more dominant animal. To incorporate this aspect of competition, we modified the test given to capuchin monkeys (*Cebus apella*) by now giving the unchosen set on every trial to a conspecific who was near the subject and who did not have to do anything to get food. Although the subjects were not directly competing for food, this manipulation should increase the competitive nature of the task for the subject animal because another individual may sometimes receive the greater quantity of food, and perhaps change the subject animal’s choice behavior during the trials with incomplete information. If it did, this would demonstrate that some social aspects of the environment can disrupt the perceptual and quantitative processing and decision-making in non-human animals, and would reflect an interaction of a “logical” decision-making process (quantity judgment with ongoing representation of average rewards) and a social factor driven by competitiveness. In that case, capuchin monkeys would respond differently in the face of the exact same quantity comparisons depending on whether a conspecific got what was left after the choice, or did not (the control condition). We were rather agnostic as to the direction of this effect (i.e., whether the monkeys should be more or less likely to choose the unknown set), but perhaps they should be less likely to choose the unknown set and potentially lose a better, and initially visible, outcome to a conspecific.

Giving the unselected food to the conspecific also meant that the focal monkeys now would see the contents of the unknown set even when they had not selected that set, and this differed from the procedure used in Beran et al. (2012). These manipulations should not have any effect during the first 15 trials, because the subject monkey would know the contents of both sets, and so should simply maximize its own reward, but in the second 15 trials, where risk was introduced and uncertainty was involved, performance might differ. At the same time, if it did not, this would demonstrate that the heuristic at work in this species (and, presumably, in humans and chimpanzees) is robust and not sensitive to disruption through this particular social manipulation or the manipulation of showing monkeys what they *did not* receive on each of these trials when they selected the known set.

## Materials and Methods

### Participants

We tested four capuchin monkeys housed at the Language Research Center (LRC). All monkeys had participated in multiple quantity judgment studies (Beran et al., 2007; Evans et al., 2009) including the previous study assessing judgments involving incomplete information (Beran et al., 2012). Each of these four focal monkeys was paired with a partner monkey that served as a passive recipient of food in the Conspecific Present condition. The focal monkeys were Wren (female), Griffin (male), Nala (female), and Liam (male), and they worked with four other monkeys (Drella, Lily, Gabe, and Logan) that only ever served to receive the free pellets from the unselected set. The capuchin monkeys were group housed but voluntarily separated for testing. Monkeys voluntarily entered individual stainless steel mesh test boxes (33 × 46 × 61 cm) that were attached to the group enclosure. There were four test boxes positioned 0.5 m apart in a row. The focal animal was always shifted into the same test box, and the partner was shifted into the same adjacent test box during the partnered condition. While there, both animals had clear visual and auditory access to one another. All other test boxes remained empty during test sessions. Water was available *ad libitum*, and all monkeys were fed manufactured chow and various fruits and vegetables daily between 1600 and 1800 h. This study complied with protocols approved by the Georgia State University IACUC. All procedures were performed in full accordance with the USDA Animal Welfare Act and conformed to the “Guidelines for the use of laboratory animals.”

### Materials

**Figure 1:**
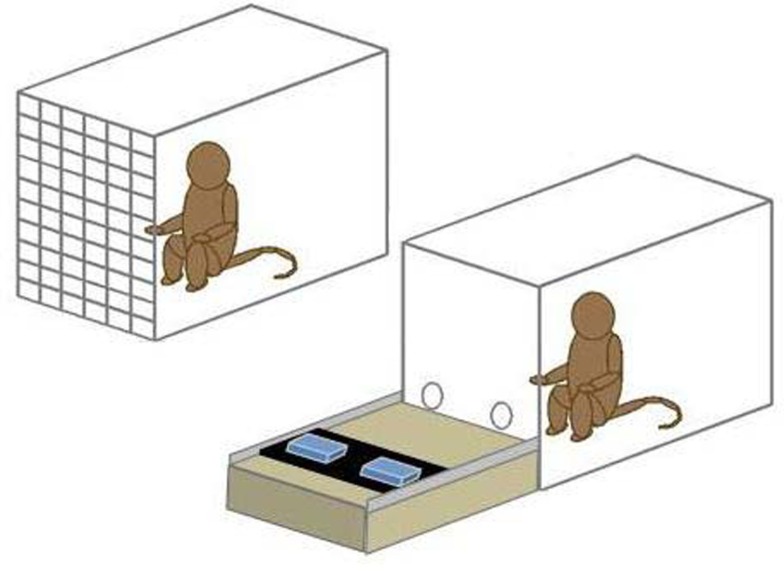
**Schematic of the test apparatus**. The box at right holds the focal monkey and the tray for presenting the stimulus sets. The conspecific, when present, was in the other box and received the contents of the non-selected set.

The apparatus consisted of a rolling cart topped with a moveable tray. The cart was positioned in front of the focal test box and the tray could be pushed toward the focal animal and pulled back to the experimenter. There were two food locations on the tray which could be covered by opaque, removable lids to conceal the contents. Focal monkeys could reach through holes in the mesh of the text box or through a Lexan cover with two arm holes to indicate their choice of one of the two food locations (see Figure 1).

### Procedures

Participants had previous experience with a similar quantity judgment task (Beran et al., 2012) and were familiar with the basic procedures of the task. In all trials of each session, Experimenter 1 baited both locations on the tray with a predetermined number of food pellets (45 mg, grain-based with banana flavor, *Bio-Serv*, Frenchtown, NJ, USA). The locations were baited out of view of the monkey by tilting the lids upward toward the experimenter, one at a time, and dropping the items all at once behind them. Each session consisted of a learning phase immediately followed by a testing phase.

For the learning phase of each session, focal monkeys were shown both sets of food items, by uncovering and recovering each set one at a time. After the presentation, the tray was pushed forward and the monkey made a choice by touching one of the lids that covered one of the sets of food items. To prevent cuing, Experimenter 1 closed his eyes and looked down while pushing the tray forward using a centrally located handle, and a second experimenter standing to the side of the apparatus (out of direct view of the monkey) announced the monkey’s choice. The focal monkey was given the amount of food under the chosen lid. Next, the unselected amount of food was shown to the focal monkey and then removed in one of two ways. In the Conspecific Absent condition, the unselected set of food items was returned to an out of sight food bowl (in the back of the cart). In the Conspecific Present condition, the unselected food was given to the partner animal in the adjacent test box, and this event occurred in full view of the focal monkey (the experimenter paused if the focal animal was not watching). It is noteworthy that, in the previous studies of this kind, the focal participant never viewed the unselected option.

Trials of the testing phase were very similar to those of the learning phase, except that the focal monkey was only shown one of the food amounts (always the amount to his or her right), instead of both amounts as in the learning phase, before being given a choice between the two options. Thus, the test phase necessarily instilled uncertainty into the quantity judgments because only one set of food items could be known with regard to its quantity. The second, unrevealed, set could be larger or smaller than the set that had been seen by the monkey. As noted earlier, these monkeys, as well as chimpanzees and humans given similar tests, relied on the approximate mean number of items received across the session’s learning phase to guide choice of the known or unknown sets in the test phase (Beran et al., 2009, 2012). When the known set was smaller than the approximate mean number of items that were obtained across the learning trials, participants previously showed a strong bias to select the unknown set (i.e., to risk the known option to try to get more food). However, when the known set was close to or larger than the mean, participants selected that set rather than the unknown option.

We tested monkeys using this procedure in three conditions.

#### Standard condition

Monkeys were given 15 learning trials and 15 test trials in each session. Four Conspecific Present and four Conspecific Absent sessions were conducted for each monkey in an alternating order. All pairwise comparisons between one and six food pellets were presented (see Table 1). For each pair of sessions (Conspecific Absent and Conspecific Present), each pairwise comparison of pellet amounts was included twice, counterbalanced for side, for a total of 30 trials and these were randomly distributed across the two sessions. The test trials in this condition consisted of the comparisons listed in Table 1, presented in random order. The number appearing in the leftmost position of each column of Table 1 indicates the number of food items shown to the monkey on every trial as the first presented set, and the number appearing in the rightmost position of each column indicates the number of food items shown in the second presented set (learning trials) or the number that was placed in the unrevealed set (test trials).

**Table 1:** **Specific quantity comparisons presented in each of the experimental conditions for each trial type**.

Standard condition		Shifting average condition		Extreme wins/losses condition	
Learning trials	Test trials	Learning trials (small set)	Test trials (small set)	Learning trials	Test trials
1,2	1,2	0,1	1,3	1,2	2,1
1,3	1,5	1,2	2,5	1,2	2,6
1,4	2,3	1,3	3,1	2,1	3,2
1,5	2,4	1,4	3,6	2,1	3,8
1,6	2,6	2,3	4,1	2,4	5,1
2,3	3,1	2,4	4,2	2,6	5,1
2,4	3,5	2,5	4,6	3,2	5,1
2,5	3,6	3,5	4,8	4,1	5,1
2,6	4,1	3,6	5,2	4,8	5,10
3,4	4,3	4,6	5,8	6,3	5,10
3,5	4,6	4,8	6,4	6,10	5,10
3,6	5,2	5,8	8,5	8,5	5,10
4,5	5,4	–	–	–	8,2
4,6	6,1	–	–	–	8,10
5,6	6,5	–	–	–	10,4

#### Shifting average condition

In the standard condition, monkeys may have come to expect on every session that the same average number of items would be obtained across trials, because the same comparisons were used in all trials, just in a different order of presentation. Thus, any potential social influences may have been diminished by a learned pattern of behavior (i.e., “always choose five items or more”). To introduce more variability into the task, we varied the average number of items that would be received in the learning phase of each session and alternated between a relatively large and small average across sessions. Twelve learning trials and 12 test trials were completed in each session. Two Conspecific Present and two Conspecific Absent sessions were completed by each monkey in a random order. The smaller average set consisted of the comparisons listed in Table 1, repeated twice and counterbalanced for side. The larger set consisted of the same comparisons multiplied by two. These test trials oversampled the middle region of values in order to provide a larger number of critical values for comparison between large-average and small-average sessions. As in the standard condition, trials were randomly ordered across a session.

#### Extreme wins/losses condition

To assess the impact of increased wins or losses after a decision, we ran a third condition in which the payout differential was more pronounced than in the previous conditions. Specifically, the critical test trials always involved the presentation of five pellets, which were paired with either 1 or 10 pellets in the non-visible set. Thus, choosing the unknown option would result in a large increase in pellets obtained or a large decrease in pellets obtained, compared to the known set. Twelve learning trials and 15 test trials were completed in each session. Three Conspecific Present and three Conspecific Absent sessions were completed by each monkey in a random order. The learning and test trials are listed in Table 1, but were randomly ordered across each session.

## Results

As would be expected from these monkeys’ past quantity judgment performance (Beran et al., 2007, 2012; Evans et al., 2009), the focal monkeys were excellent in choosing the larger of the two sets of food items when they saw both, during the first trials of each session. Performance in the training phases of all conditions is shown in Table 2. Performance was very high in all cases, and rarely differed between the Conspecific Present and Conspecific Absent conditions.

**Table 2:** **Percentage of trials selecting the larger quantity by each monkey during training trials in each condition**.

	Griffin	Wren	Liam	Nala
Standard
Conspecific present	90.0	86.67	93.3	93.3
Conspecific absent	90.0	90.0	91.67	90.0
Shifting average
Conspecific present	95.83	87.5	100	100
Conspecific absent	87.5	87.5	95.83	91.67
Extreme wins/losses
Conspecific present	83.3	86.1	94.4	97.2
Conspecific absent	83.3	86.1	91.67	94.4

**Figure 2:**
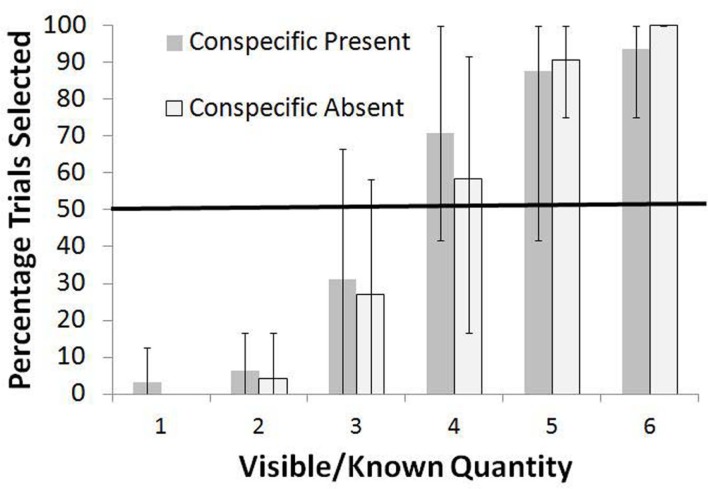
**Overall percentage of trials in which the known set was selected when paired with an unknown set in the Standard test**. Data are shown for all monkeys combined and are separated at each known quantity into the Conspecific Present and Conspecific Absent conditions. Bars show the range of responses across the monkeys.

The results for the Standard Condition are shown in Figure 2 as the total percentage of trials for all four monkeys in which the known set was selected. During the training trials, perfect performance would have led to an average of 4.4 pellets per trial. The monkeys consistently rejected three or fewer items in the known set and instead selected the unknown option at levels significantly higher than chance, all *p* < 0.05, binomial tests (these and all further binomial tests were two-tailed). For four items, they were indifferent between the two choices in the Conspecific Absent condition (*p* > 0.05, binomial test) but significantly preferred the known set in the Conspecific Present condition (*p* < 0.05, binomial test). For more than four items, they preferred the known set at levels greater than chance, all *p* < 0.05, binomial tests. Chi square tests for independence showed no difference in the frequency of selection of the known set between the Conspecific Present and Conspecific Absent conditions for any of the known quantities [all χ^2^ (df = 1) <1.70, *p* > 0.05]. Thus, there was no effect of unselected sets going to the conspecific or not.

**Figure 3:**
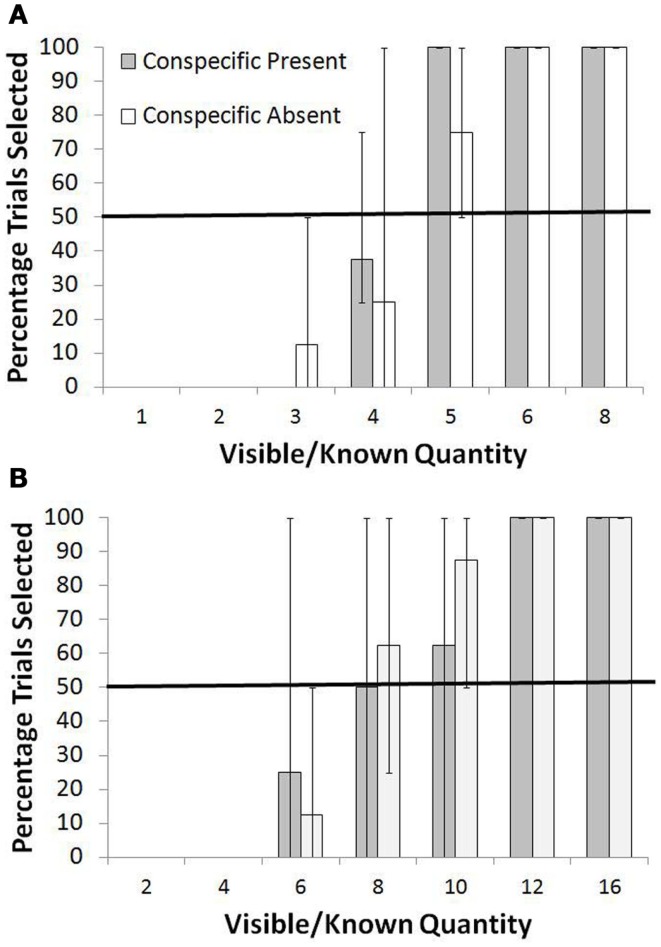
**Overall percentage of trials in which the known set was selected when paired with an unknown set in the Shifting Average test**. **(A)** Shows performance with the smaller range of quantities, and **(B)** shows performance with the larger range of quantities. Bars show the range of responses across the monkeys.

The results for the Shifting Average Condition are shown in Figure 3A for the smaller range and in Figure 3B for the larger range. During the training trials for the smaller range, perfect performance would have led to an average of 4.67 pellets per trial. Because of the lower trial counts, we combined some of the known quantity values to contrast low values, intermediate values, and high values. For test trials in the smaller range, the monkeys consistently rejected three or fewer items in the known set and instead selected the unknown option at levels significantly higher than chance, *p* < 0.05, binomial test. For four items, they were indifferent between the two choices in the Conspecific Present condition and the Conspecific Absent condition (*p* > 0.05, binomial test). For more than four items, they showed a preference for the known set, selecting that set on 16 of 16 trials in the Conspecific Present condition and 14 of 16 trials in the Conspecific Absent conditions, both *p* < 0.01, binomial test. A chi square test for independence showed no difference in the frequency of selection of the known set between the Conspecific Present and Conspecific Absent conditions across all known quantities [all χ^2^ (1, *N* = 48) <1.00, *p* > 0.05].

For the larger range, the pattern was similar. During the training trials for the larger range, perfect performance would have led to an average of 8.16 pellets per trial. In test trials of the larger range, the monkeys preferred the unknown option in both Conspecific Present and Conspecific Absent conditions when the known option now was six items or less (both *p* < 0.01, binomial tests), they were indifferent when the known option had eight items (both *p* > 0.05, binomial tests), and they preferred the known set when it had more than eight items (both *p* < 0.05, binomial tests). And, again, there was no difference in the frequency of selection of the known set between the Conspecific Present and Conspecific Absent conditions across the known quantities [allχ^2^ (1, *N* = 48) <1.00, *p* > 0.05].

The two quantity ranges in this part of the experiment shared three common quantities (four, six, and eight) that were presented as the known set, and the choice of those quantities was significantly different depending on whether they were presented as part of the small or large range. For the small range, known sets with four items were chosen more often than for the large range, χ^2^ (1, *N* = 64) = 4.06, *p* < 0.05. This was also true for known sets of six items, χ^2^ (1, *N* = 24) = 14.18, *p* < 0.05, and for known sets of eight items, χ^2^ (1, *N* = 40) = 5.38, *p* < 0.05.

**Figure 4:**
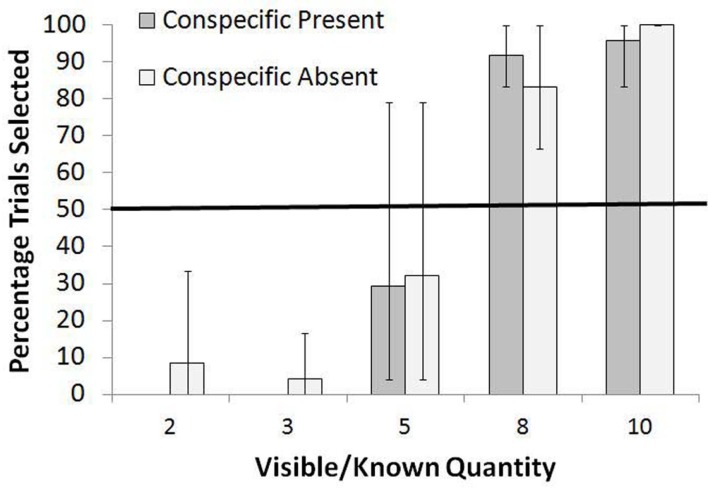
**Overall percentage of trials in which the known set was selected when paired with an unknown set in the Extreme Wins/Losses test**. Bars show the range of responses across the monkeys.

For the Extreme Wins/Losses Condition, the mean number of items obtained if perfect during the training trials was 5.5 items. The results for this condition are shown in Figure 4. The monkeys consistently rejected five or fewer items in the known set and instead selected the unknown option at levels significantly higher than chance, all *p* < 0.05, binomial tests. For more than five items, they preferred the known set at levels greater than chance, all *p* < 0.05, binomial tests. Once again, there was no difference in the frequency of selection of the known set between the Conspecific Present and Conspecific Absent conditions for any of the known quantities [all χ^2^ (df = 1) <1.00, *p* > 0.05].

## Discussion

All four focal monkeys performed in a similar manner, and replicated their performance in the earlier experiment on estimating uncertain outcomes in a quantity judgment task (Beran et al., 2012). They approached the trials with incomplete information by responding on the basis of using the approximate average number of items they had received to that point in the test session (during the training trials). If the known quantity was smaller than that amount, they generally gave up that option and instead took the unknown quantity. If the known amount was as large as, or larger than, the average, they selected it. As in the previous studies of this type (Beran et al., 2009, 2012), this performance was not based on some absolute value that was always the threshold for selecting the known set. In the Shifting Average condition, where we could double or halve the average from session to session, the monkeys changed their threshold to accompany those changes. This means that the monkeys did shift their indifference point across the two ranges, in a manner consistent with continued use of the average number of items obtained in training (4.67 for the smaller range and 8.16 items for the larger range if one assumes every training trial was completed correctly). This demonstrated their flexibility in using the heuristic to deal with unknown and incomplete information.

As with the data reported by Beran et al. (2009), it is important to note that the arithmetic mean is not the only measure of central tendency that might be used by non-human animals in this kind of situation. It is sometimes reported that in tests of quantity estimation or comparison that animals’ responses are best accounted for by use of the geometric mean (the square root of the product of the anchor values; Roberts, 2005; Jordan and Brannon, 2006; Beran et al., 2008). For the specific quantities in each range we used in this experiment, the geometric means and the arithmetic means were quite similar (range 1–6: geometric mean = 2.99, arithmetic mean = 3.5; range 1–8: geometric mean = 3.44, arithmetic mean = 4.14; range 2–16: geometric mean = 6.89, arithmetic mean = 8.28). Thus, it is difficult to determine which measure of central tendency might have been used by the animals. Future research will be needed to better establish this.

What was novel in the present experiment was the introduction of a social component to the test, and a highly salient one in terms of the task setup. Now, on half of the sessions, the focal monkey watched as its unchosen set was given to a conspecific, who was allowed to eat those pellets in full view of the focal monkey. Although during training trials the focal animal nearly always got the larger amount, the monkeys still observed and attended to the smaller amount being given to the conspecific. Also, during trials with incomplete information, it was possible for the partner animal to get the larger amount, as when a known set was selected by the focal animal but the unknown set was larger, or when the focal monkey selected an unknown set that turned out to be smaller than the known set.

It was also a new manipulation that the focal monkey now got to see the unchosen set on test trials where it took the known quantity. In the past, the monkeys never knew what they forewent in making their selection in the uncertain trials (Beran et al., 2012), whereas here they could see whether choosing the known quantity ended up being a good choice, or a bad one, in terms of the amount of food in the unknown set. However, these new aspects to the methodology appeared to have no effect on the decisions made by focal monkeys, at least as they pertained to the choice behavior. However, what is not clear is whether seeing food items given to other animals might change the “running average” held by a subject in other circumstances. For example, the monkeys may have reacted differently if the set given away was unexpectedly larger than would have been predicted by what had occurred to that point in the session. Perhaps more extreme outcomes, coupled with the social manipulation, would change the performance of monkeys in making these judgments.

Putting animals in tests in which there is actual competition, the appearance of competition, or even just situations in which conspecifics are given food for the efforts of the subject, can change the behavior and performance of the focal subject (e.g., Washburn et al., 1990). This can be true even at the level of judging the perspective of others. For example, chimpanzees and rhesus monkeys seemed to respond differently in judging other animals’ visual knowledge when placed in a competitive task versus a non-competitive task (Tomasello and Call, 1997; Povinelli, 2000; Hare et al., 2001; Flombaum and Santos, 2005). This effect, however, does not seem to occur for perception of quantity, even in contexts in which judgments about the likelihood of getting more food for taking a risk occurs. Rather, the capuchin monkeys in this experiment, when faced with incomplete information, seemed to disregard the presence or absence of a conspecific that received whatever the subject did not choose. Instead, the monkeys sustained what appeared to be an optimal heuristic response in using the average number of pellets they had been receiving up to that point in the session as a threshold for making choices when they could not know both sets. Prior experience from earlier studies along with information feedback may have impacted the monkeys’ reliance on the heuristic, potentially overshadowing any deleterious effects of a competitive-like situation. Thus, perception and decision-making in a quantity judgment task appear to be insulated from any negative effects of a more competitive test environment, although other more overt manipulations to an animal’s social environment might yet evoke less optimal responding.

## Conflict of Interest Statement

The authors declare that the research was conducted in the absence of any commercial or financial relationships that could be construed as a potential conflict of interest.
